# Weapons against Themselves: Identification and Use of Quorum Sensing Volatile Molecules to Control Plant Pathogenic Fungi Growth

**DOI:** 10.3390/microorganisms10122459

**Published:** 2022-12-13

**Authors:** Caroline De Clerck, Laurie Josselin, Valentine Vangoethem, Ludivine Lassois, Marie-Laure Fauconnier, Haïssam Jijakli

**Affiliations:** 1 AgricultureIsLife, Gembloux Agro-Bio Tech, Liege University, Passage des Déportés 2, 5030 Gembloux, Belgium; 2 Laboratory of Chemistry of Natural Molecules, Gembloux Agro-Bio Tech, Liege University, Passage des Déportés 2, 5030 Gembloux, Belgium; 3 Integrated and Urban Plant Pathology Laboratory, Gembloux Agro-Bio Tech, Liege University, Passage des Déportés 2, 5030 Gembloux, Belgium; 4 Plant Genetics and Rhizosphere Processes Lab., Gembloux Agro-Bio Tech, Liege University, Passage des Déportés 2, 5030 Gembloux, Belgium

**Keywords:** quorum sensing, *Fusarium culmorum*, *Cochliobolus sativus*, volatile organic compounds (VOCs), antifungal

## Abstract

Quorum sensing (QS) is often defined as a mechanism of microbial communication that can regulate microbial behaviors in accordance with population density. Much is known about QS mechanisms in bacteria, but fungal QS research is still in its infancy. In this study, the molecules constituting the volatolomes of the plant pathogenic fungi *Fusarium culmorum* and *Cochliobolus sativus* have been identified during culture conditions involving low and high spore concentrations, with the high concentration imitating overpopulation conditions (for QS stimulation). We determined that volatolomes emitted by these species in conditions of overpopulation have a negative impact on their mycelial growth, with some of the emitted molecules possibly acting as QSM. Candidate VOCs related to QS have then been identified by testing the effect of individual volatile organic compounds (VOCs) on mycelial growth of their emitting species. The antifungal effect observed for the volatolome of *F. culmorum* in the overpopulation condition could be attributed to ethyl acetate, 2-methylpropan-1-ol, 3-methylbutyl ethanoate, 3-methylbutan-1-ol, and pentan-1-ol, while it could be attributed to longifolene, 3-methylbutan-1-ol, 2-methylpropan-1-ol, and ethyl acetate for *C. sativus* in the overpopulation condition. This work could pave the way to a sustainable alternative to chemical fungicides.

## 1. Introduction

Microbes are known to be able to communicate with each other using chemical signals through a phenomenon known as quorum sensing (QS) [1]. QS is often defined as a mechanism of microbial communication that can regulate microbial behaviors in accordance with population density. This cell to cell signaling is mediated through the emission of small molecules that diffuse in the extracellular environment and accumulate during the growth of the microbial population [2]. These molecules, called auto-inducers (able to stimulate their own production) or quorum sensing-molecules (QSM), are usually released continuously and their concentration increases proportionally to the microbial population [3]. When a critical threshold is reached, a number of cell density-related regulatory responses are triggered in a coordinated manner [4,5]. These mostly involve the expression or repression of specific QS-dependent target genes in the whole population. QS mechanisms are thought to allow the coordination of the physiology of the community, giving an advantage for adaptation to the rapidly changing conditions of an environment [3].

While fungal QS research is still in its infancy, a lot of studies have been made on bacteria, where QS is known to be involved in the regulation of processes such as pathogenesis, symbiosis, competence, conjugation, nutrient uptake, morphological differentiation, secondary metabolite production, and biofilm formation [2,5].

Many behaviors that are dependent on cell density, and thus potentially regulated by QS, have also been described in fungi, but the molecules and pathways involved in those processes remain unknown at this time.

QS investigation in eukaryotes began with the discovery of farnesol, tyrosol, and phenylethanol, which are all produced as QSM by the human fungal pathogen *Candida albicans* [6,7]. In the following years, more fungal species have been studied for their QS activities and/or the effect of inoculum size: *Histoplasma capsulatum* [8], *Ophiostoma ulmi* [9], *Neurospora crassa* [10], *Saccharomyces cerevisiae* [11], *Cryptococcus neoformans* [12], *O. floccosum* [13], *Aspergillus nidulans* [14], *A. flavus* [15], *Penicillium sclerotiorum* [16], *Fusarium oxysporum* [17], *P. marneffei* [3], *O. piceae* [3], *Mucor rouxii* [18], *P. isariaeforme* [19], and *Schizosaccharomyces japonicus* [20].

Until today, several types of QSM have been identified in fungi: pheromone peptides, aromatic alcohols, terpenes, lactone containing molecules, and lipids (oxylipins) [21,22]. Several studies have also reported that QSMs could be volatile organic compounds [22,23,24,25].

As in bacteria, QS regulation is thought to allow fungi to better adapt to harsh environments or to be involved in interactions with various hosts [26]. It also contributes to eukaryote specific reproductive strategies or molecular or cellular events [26]. In fungi, QS mechanisms have been described to regulate processes such as sporulation, secondary metabolite production, morphological transition, enzyme secretion, biofilm assembly, and apoptosis. They may also act as antimicrobial agents [27].

Several scientists agree that QSM appears to be a promising way to achieve new methods to manage fungal infections and eventually multidrug resistance in the case of widely antifungal treatments [3,26]. In our study, we focus on two plant pathogenic fungal species that have never been investigated for QS before: *Cochliobolus sativus* and *Fusarium culmorum*. These pathogens are causing agents of several important diseases in cereals (mostly wheat and barley for *C. sativus*, with *F. culmorum* having a wider variety of hosts), leading to significant reductions in grain yield and quality [28,29].

Both fungi are associated with the common root rot of barley. This disease of agronomical importance can cause between 9% and 23% yield losses, depending on growth area and cultivars [30,31].

The aims of the present work are therefore:

- To determine the molecules constituting the volatolome of *F. culmorum* and *C. sativus* during culture conditions involving low and high spore concentrations.
- To determine whether the volatolome of each fungus in overpopulation conditions has an impact on the mycelial growth of the same species in low spore culture conditions.
- To identify in these volatolomes potential QSM as a first step in the development of a plant fungal pathogen control method using VOCs produced by the targeted species.

## 2. Materials and Methods

### 2.1. Biological Material

Fungal strains used are *Fusarium culmorum* Schltdl. and *Cochliobolus sativus* Drechsler ex Dastur. They are from the Belgian coordinated collections of microorganisms (BCCM-MUCL, Ottignies-Louvain-la-Neuve, Belgium) where they are recorded as MUCL 28166 and MUCL 46854, respectively.

Fungi were cultivated on Potato Dextrose Agar (PDA) medium (Merck KGaA, Darmstadt, Germany, 39 g.L^−1^) at 23 °C and a 16:8 (L:D) photoperiod. Cultures were used after 14 days of growth to make spore suspensions.

### 2.2. Collection and Identification of Fungal Volatile Organic Compounds (VOCs)

#### 2.2.1. Sample Preparation

Cultures were made in Solid Phase Microextraction glass vials of 20 mL (Filter Service, Eupen, Belgium). Prior to the culture, vials were washed for 1 h in distilled water containing 1% of RBST 150 detergent (Chemical Products R. Borghgraef S.A, Brussels, Belgium) then rinsed three times with distilled water and one time with ethanol before being placed in an oven at 120 °C for 1 h. Once dry, the vials were filled with 7 mL of PDA medium and autoclaved. They were then allowed to cool in an inclined position to obtain a slope in the culture medium. Vials were then inoculated with 50 µL of spore suspensions for each treatment and placed at 23 °C and a 16:8 (L:D) photoperiod.

Inoculations were performed either with spore suspensions of 2 × 10^3^ spores.mL^−1^ (low spore concentration, called normal growth conditions in this study; this suspension’s concentration permited growth without high competition) for both fungi, 10^7^ spores.mL^−1^ (high spore concentration; this causes overpopulation conditions for *F. culmorum*), and 10^6^ spores.mL^−1^ (high spore concentration; this causes overpopulation conditions for *C. sativus*).

Two growing durations were tested: 7 and 14 days. For each treatment (*F. culmorum* and *C. sativus* each in overpopulation and normal growth conditions), three repetitions were carried out. One empty vial and one vial containing only PDA were also analyzed for each condition.

#### 2.2.2. Extraction of VOCs Emitted by Both Fungi in Normal and Overpopulation Conditions

Solid Phase Micro Extraction (SPME) fibers coated with three 65 µm thick stationary phases: divinylbenzene-carboxen-polydimethylsiloxane (DVB-Carboxen-PDMS) (Supelco, Bellefonte, PA, USA) were used. All fibers were conditioned before first use at 270 °C for 1 h according to the supplier’s recommendations.

Before VOC sampling, vials were equilibrated for 10 min at 25 °C in a water bath; the SPME fibers were then inserted into the headspaces for 20 min at the same temperature.

#### 2.2.3. Analysis of VOCs Emitted by Both Fungi in Normal and Overpopulation Conditions

Gas chromatography–mass spectrometry (GC-MS) analyses followed the protocol described in Fiers et al. [30] and were performed on a gas chromatograph (Agilent Technologies 7890A, Santa Clara, CA, USA) coupled to a mass selective detector (Agilent Technologies 5975C) with enhanced Chemstation version E.02.00.493 software. The GC, fitted with a Gerstel MPS autosampler (Maestro 1, version 1.4.8.14/3.5; Müllheim/Ruhr, Germany), was used for SPME injection. After extraction, the volatile compounds were desorbed in the pulsed splitless mode for 5 min at 250 °C. Separation was performed on a VF-WAX column (Agilent Technologies, USA, 30 m × 0.250 mm I.D, 0.25 µm film thickness). Helium was used as a carrier gas at a constant flow rate of 1.5 mL.min^−1^. The inlet temperature was 250 °C. Pulsed splitless injection mode in a 1.5 mm HS-liner was used (injection pulse pressure of 30 psi for 1 min). The following temperature programs were used: 35 °C for 2 min, 5 °C.min^−1^ up to 155 °C, 20 °C.min^−1^ up to 250 °C, and a final hold at 250 °C for 10 min. The mass spectrometer was operated in electron ionization (EI) mode at 70 eV, source temperature 230 °C, quadrupole temperature 150 °C, scanned mass range from 20 to 350 amu, threshold of 150 amu, and a scan speed of 4.27 scans.s^−1^.

#### 2.2.4. Identification of VOCs Emitted by Both Fungi in Normal and Overpopulation Conditions

Identification was made by comparing retention times and recorded mass spectra using the Wiley 275, pal600k and NBS75K spectral databases. In addition, the theoretical non-isothermal Kovats retention indices of each molecule found in the literature were compared with calculated retention indices. Retention indices were calculated by injecting saturated n-alkane standard solution C7–C30 (1000 µg.mL^−1^ in hexane, Supelco, Overijse, Belgium), using the definition of Van den Dool and Kratz [32]. For seven compounds of interest (ethyl acetate, toluene, 3-methylbutyl ethanoate, 3-methylbutan-1-ol, pentan-1-ol, (+)-sativene, and longifolene), identifications were confirmed by injecting commercial standards provided by Sigma-Aldrich (St. Louis, MO, USA) with the highest chromatographic purity. A mix was made from pure concentrated standards by diluting standards 10^3^-fold in ethanol. For GC-MS measurements, 1 mL of a standard mix was placed inside a 20 mL vial before quickly sealing it securely. Extraction was then made following the same protocol than described earlier. Three repetitions were made for each standard.

### 2.3. Characterization of the Effect of Fungal Volatolomes on Fungal Growth

**Figure 1:**
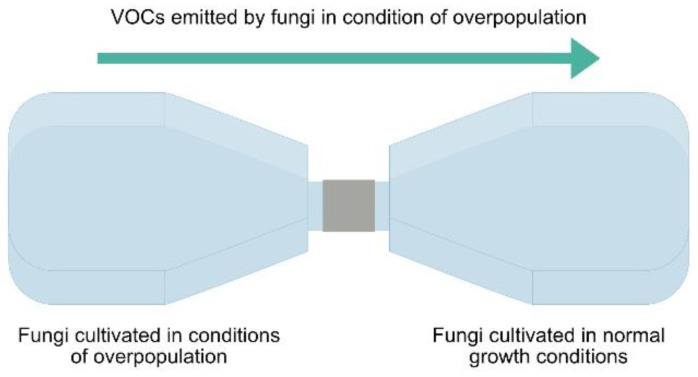
Experimental setup used to observe the effect of the fungal volatolome on fungal growth without physical contact.

The experimental setup was built using two cell culture flasks of 600 mL (Greiner Bio-One, Vilvoorde, Belgium) put end to end and hermetically secured on the outside with tape and parafilm (Figure 1). In this way, no direct contact could occur between both flasks, but VOCs could freely circulate. Fifty milliliters of autoclaved PDA medium were placed in each flask before the sealing. The first flask contained the fungus in overpopulation, inoculated using 0.5 mL of a spore suspension of 10^7^ spores.mL^−1^ for *F. culmorum* and 10^6^ spores.mL^−1^ for *C. sativus.* The second flask contained the fungus of the same species, inoculated using a 5 mm mycelium disc to get a circular and measurable growth. For each fungus, a negative control was carried out, in which the first flask contained the fungus in normal growth conditions, and the second flask only PDA. Eight repetitions were conducted for each combination. Flasks were placed at 23 °C and a 16:8 (L:D) photoperiod. The mycelial growth diameters of the fungi cultured in normal growth conditions were measured daily for 14 days (or until the mycelium filled the flask).

### 2.4. Characterization of Individual VOCs’ Effects on Fungal Growth

**Figure 2:**
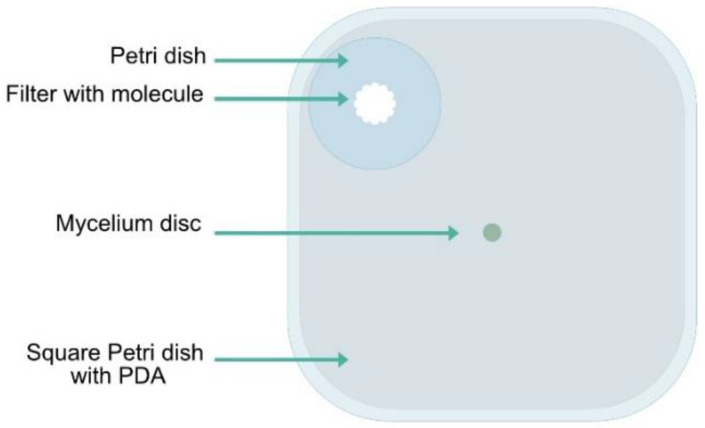
Experimental setup used to observe the effect of individual VOCs on fungal growth.

The experimental setup was made of a square 12-cm Petri dish (Greiner, Vilvoorde, Belgium), containing the lid of a small round Petri dish (36.5 mm diameter) in a corner. Fifty milliliters of PDA medium was poured inside the square Petri dish (Figure 2). When the medium was solidified, the fungus (either *F. culmorum* and/or *C. sativus*) was inoculated on the PDA medium using a 5 mm mycelium disc. Each VOC to be tested was then placed on a filter paper positioned in the small Petri dish. Both parts of the placed articles were thus not in direct contact but share the same atmosphere, enriched in the tested individual VOC.

Eight compounds were chosen to perform the following tests. Some are emitted by both strains studied, other are strain specific (Table 1). Four concentrations were tested for each molecule (0 as negative control, 100, 500, and 1000 µM), except for (+)-sativene which was tested only at 500 and 1000 µM and for the 3-methylbutyl ethanoate tested with 1000, 1500, and 2000 µM. The tested compounds are found in liquid form. The volume placed on the filter was calculated using following formula:

(1) $$V_{to\;collect}=\frac{C_{compound}.\;M.V_{air\;Petri\;dish}}{molecular\;density\;}$$

where *M* is the molar mass of the molecule, *Ccompound* the concentration wanted for the molecule, *V_air Petri dish_* the volume or air contained in the square petri dish (0.1 L). Four repetitions were made by concentration and by fungal species. The square Petri dishes were hermetically closed with a double layer of parafilm and placed in a culture chamber at 23 °C and a 16:8 (L:D) photoperiod. The mycelial growth diameters of the fungi were daily measured for 14 days, and the factor of mycelial growth reduction was established using the formula below.

(2) $$Factor\;of\;growth\;reduction=\frac{Diameter_{sample}-Diameter_{control}}{Diameter_{control}\;}$$

### 2.5. Statistical Analysis

Results were statistically processed with Minitab17.1.0^®^ software. One-way ANOVA and Dunnett’s mean structuring were used. This Dunnett’s method allows a comparison of the effects of several modalities of a treatment with a reference level. In this study, the baseline will be controls performed for each treatment and each analysis.

## 3. Results

### 3.1. Collection and Identification of Fungal Volatile Organic Compounds

**Figure 3:**
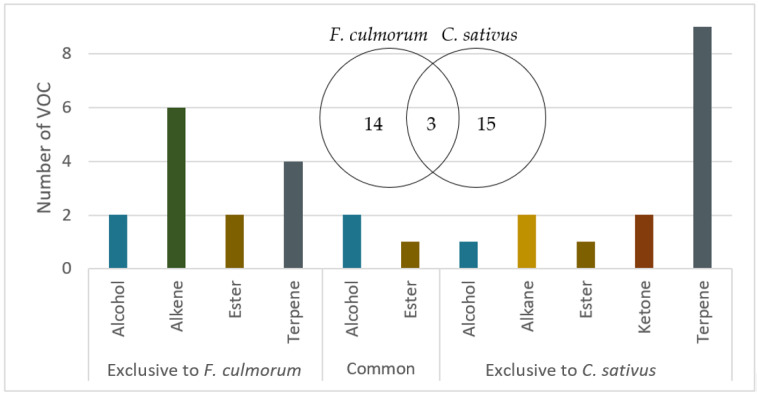
Number of VOCs emitted by *F. culmorum* and *C. sativus* distributed by chemical family (independent of the two culture conditions).

A total of 32 VOCs were identified for both species and growth conditions combined (Table 2). The comparison of VOCs emitted by the two species, all growth conditions considered, shows a common base of three VOCs (ethyl acetate, 3-methylbutan-1-ol, and 2-methylpropan-1-ol) (Figure 3). Differences in the chemical families of VOCs associated exclusively with one of the two species are notable. Indeed, a greater number of alkenes are emitted by *F. culmorum* whereas VOCs of the terpene family are predominantly emitted by *C. sativus*. In addition, ketones are only detected for the *C. sativus* species (Figure 3).

**Figure 4:**
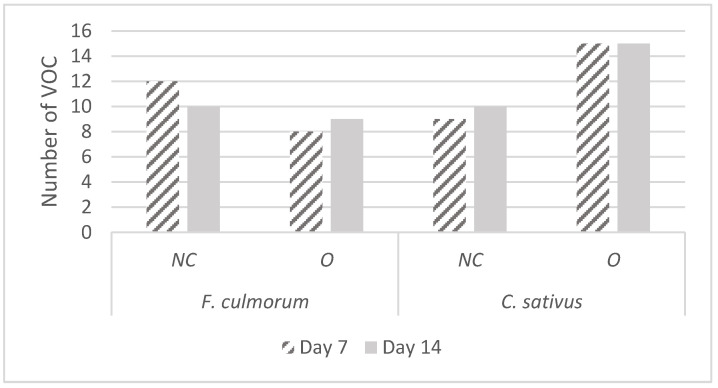
Number of VOCs emitted by *F. culmorum* and *C. sativus* after 7 and 14 days of culture in conditions of normal (NC) and overpopulation (O) growth.

In addition, we observed that *F. culmorum* was emitting a greater number of VOCs during normal conditions of growth (low spore concentration) than when it was in overpopulation (high spore concentration). The opposite trend was observed for *C. sativus* (Figure 4).

**Figure 5:**
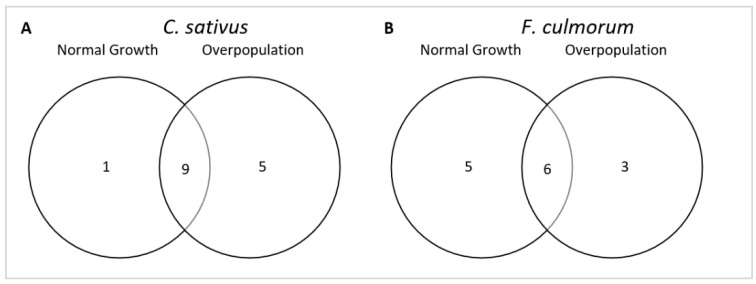
Grouping the specific VOCs emitted by each species in the normal and overpopulation growth conditions. (**A**) VOCs emitted by *C. sativus* (**B**) VOCs emitted by *F. culmorum*.

Among the 15 specific VOCs emitted by *C. sativus*, nine were commonly emitted under both growth conditions, five were emitted only under overpopulation conditions (propan-1-ol, ethyl 2-methylbutyrate, acetoin, butan-2-one, and unknown sesquiterpene 1), and one (α-gurjunene) was only emitted under normal growth conditions (Figure 5A). Concerning the 14 VOCs specific to *F. culmorum*, six were common to both growth conditions, five VOCs were only emitted in the case of normal fungal growth conditions (β-phellandrene, p-xylene, mesitylene, 2,6-dimethylanisole, and 2-phenylethanol), and three in conditions of overpopulation (pseudocumene, hemellitol, and pentan-1-ol) (Figure 5B).

### 3.2. Characterization of the Fungal Volatolome Effect on Fungal Growth

**Figure 6:**
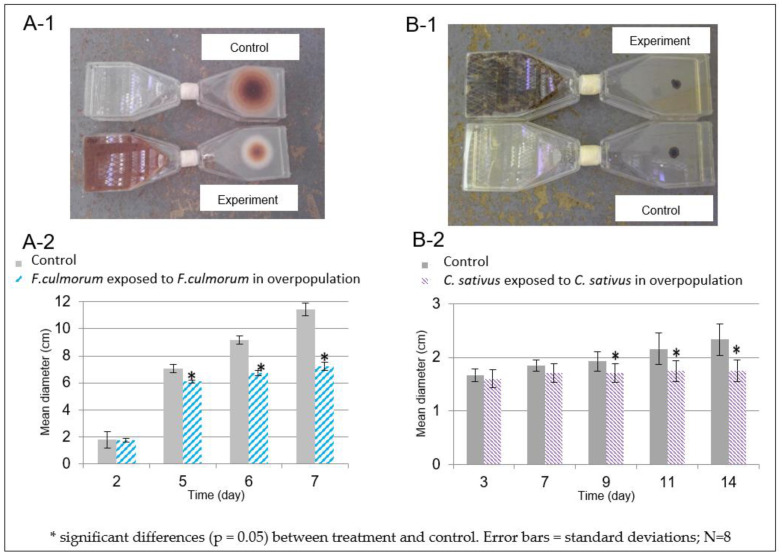
Pictures and effects of *F. culmorum* and *C. sativus* volatolomes under overpopulation conditions towards *F.culmorum* (**A**) or the *C. sativus* (**B**) growth in normal condition.

In our experiments, we observed that the growth of *F. culmorum* exposed to the volatolome coming from *F. culmorum* grown in overpopulation was reduced in comparison to the non-exposed negative control, with its mycellium being less developed and pigmented (Figure 6A-1). The measured differences were confirmed to be statistically significant after 5 days of growth (Figure 6A-2).

For *C. sativus*, no visual differences were observed using the same dispositive (Figure 6B-1). Measurements, however, showed a significant decrease from day 9 of growth. This decreasing trend was maintained until the end of the experiment (Figure 6B-2).

### 3.3. Characterization of VOCs’ Individual Effects on Fungal Growth

Several VOCs produced by *F. culmorum*, *C. sativus,* or both have been individually tested on the fungal species that were emitting them to determine their impact on fungal growth and their possible involvement in quorum sensing mechanisms.

**Figure 7:**
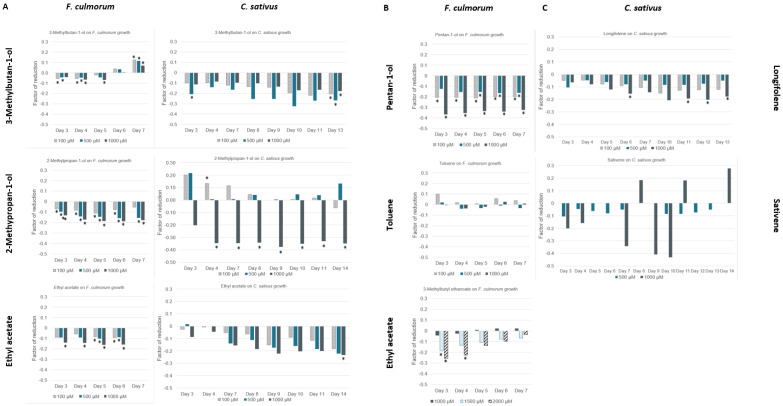
Factor of reduction of the growth of *F. culmorum* and *C. sativus* in the presence of individual VOCs at different concentrations over time (**A**): VOC tested commonly against the two species; (**B**): only against *F. culmorum*; (**C**): only against *C. sativus*. * Means statistically significant results.

Three of the tested VOCs were emitted by both fungal species of this study (Figure 7A): 3-methylbutan-1-ol induced a significant growth reduction from day 3 for both fungi. However, whereas a continuous reduction was maintained until the 13th day of growth for *C. sativus,* a slight growth stimulation (+0.1) was observed for *F. culmorum* from day 6, and this effect increased with time.

2-methylpropan-1-ol was shown to significantly reduce *F. culmorum* growth for the entire growing period. For *C. sativus*, two different trends were observed depending on the concentration applied: at 100 µM, *C. sativus* growth was first stimulated (days 3 to 8), but this effect tended to disappear with time and a trend to growth reduction began to appear on day 14. On the other hand, at 1000 µM, a significant growth reduction is observed from day 3 until day 14.

For ethyl acetate, a significant growth reduction was observed for *F. culmorum* all throughout the growth period at 1000 µM, while only from day 5 of growth for lower concentrations. In the case of *C. sativus*, a trend to growth reduction was observed for all concentrations, but results were not significant due to high variability.

Concerning the two VOCs specifically emitted by *C. sativus*, we observed that longifolene induced a strong reduction in growth, with a peak at day 11 and 12 for the highest concentration. For (+)-sativene, results were variable, with alternations in growth increase and reduction; no significant value was reported.

In the case of *F. culmorum*, for toluene, no significant difference in growth were observed in comparison to the non-treated control. For 3-methylbutyl ethanoate, an antifungal effect was observed from concentrations above 1500 µM the first 4 days of growth. However, the effect tended to decrease over time. Finally, pentan-1-ol induced a strong growth reduction on *F. culmorum* at all concentrations; this effect remained constant over time.

In brief, the most interesting antifungal effects were obtained with pentan-1-ol for *F. culmorum* at the three concentrations tested and 2-methylpropan-1-ol for *C. sativus* at 1000 µM.

## 4. Discussion

The literature on VOCs detected for fungi in conditions of overpopulation and/or QS is still very sporadic and focused on other species. This work puts forward, for the first time, the VOCs differentiating between two types of growth conditions for *C. sativus* and *F. culmorum*.

In this study, we have observed that the total number of VOCs emitted by each species for each growth condition was constant over time. However, the number was different between the two growth conditions, with *F. culmorum* releasing more VOCs under normal growth conditions than in overpopulation, while the opposite was observed for *C. sativus* (Figure 3). These differences are not surprising, as several papers have already mention the fact that the volatolome of a defined fungal strain can be influenced by several parameters (age of culture, environmental conditions, exposure to other fungal VOCs…) [36,37], but very few studies have compared the whole volatolome of the same strain in normal and overpopulation conditions, and have instead mainly focused on single compounds [36].

In all growth conditions combined, we have detected the specific compounds known to be emitted by the two fungal species, validating our sampling method: germacrene A, (+)-sativene, and isoprene for *C. sativus* [30] and epi-bicyclosesquiphellandrene, β-phellandrene [33], and p-xylene for *F. culmorum* [35].

In addition, in normal growth conditions, we have detected new compounds that have not been specifically associated to the two species in the literature: 2,6-dimethylanisole, mesitylene, and 2-phenylethanol for *F. culmorum*, and α-gurjunene for *C. sativus* (Table 2).

However, all these compounds have been detected to be emitted by other fungal species [38,39,40,41]. Interestingly, 2-phenylethanol has been shown to have antifungal properties against Penicillium species [41].

In overpopulation conditions, three new specific VOCs (pentan-1-ol, hemellitol, and pseudocumene) were detected for *F. culmorum* while five (propan-1-ol, ethyl 2-methylbutyrate, acetoin, butan-2-one, and unknown sesquiterpene 1) were found to be specifically released by *C. sativus*. We cannot exclude that these compounds were also produced in normal growth conditions in quantities below the quantification limits of our detection method. QSM molecules are indeed continuously emitted by microorganisms and accumulate in parallel with the population growth. To our knowledge, none of these compounds have been identified in the literature to be associated with overpopulation or QS in fungi.

It is interesting to note that three of the trimethylbenzene isomers were detected for *F. culmorum,* but not all for all growth conditions (mesitylene was detected in normal growth condition, while pseudocumene and hemellitol in overpopulation). A hypothesis could be that a change in the metabolic pathway or the activation of an enzyme has led to the modification of mesitylene into one of the other two isomers. While pseudocumene and hemellitol are two isomers with reported toxic properties, mesitylene is not [42].

Ethyl 2-methylbutyrate has been identified to be emitted by non-toxigenic strains of *Aspergillus flavus* [43].

Acetoin is known to be produced by plant-associated *Enterobacteriaceae* and is involved in the pathogenesis of some plant pathogen species such as *Pectobacterium carotovorum* [44]. Several hypotheses could be associated with its detection in *C. sativus*. It could be the result of a modification of a biological function linked to the depletion of the carbon source because acetoin can be a source of carbon and energy [45]. Some studies on *B. subtilis* [46] have also shown that acetoin could be emitted during biofilm induction.

Our work showed that the volatolomes of *F. culmorum* and *C. sativus* grown under overpopulation conditions caused a significant growth reduction on the species they were emitted from. This effect was shown to be significant from 5 and 11 days of growth for *F. culmorum* (Figure 6A-2) and *C. sativus* (Figure 6B-2) respectively.

Therefore, eight compounds from these volatolomes were selected and tested separately on the growth of the species that produced them. The selected compounds were released at 7 days of growth, were part of the main peaks found in the chromatograms, and presented a good identification quality. They also have been selected according to the inhibitory potential found in the literature and their commercial availability.

The antifungal effect observed for the volatolome of *F. culmorum* in overpopulation coniditions could be attributed to ethyl acetate, 2-methylpropan-1-ol, 3-methylbutyl ethanoate, 3-methylbutan-1-ol, and pentan-1-ol. The greatest reduction in growth was observed with pentan-1-ol (Figure 7B).

However, the individual VOCs had a smaller effect than the whole volatolome, demonstrating that a synergy of VOCs is likely at the origin of this growth reduction. Another possibility could be that other compounds of the volatolome not individually tested (like hemellitol and pseudocumene, known for their toxic properties [42]), largely contribute to the growth reduction.

In the same way, the antifungal effect observed for the volatolome of *C. sativus* in overpopulation could be attributed to longifolene, 3-methylbutan-1-ol, 2-methylpropan-1-ol, and ethyl acetate (Figure 7A). Unlike with *F. culmorum*, the total growth reduction obtained for the combination of these molecules was equal to the reduction observed with the whole volatolome.

Some of these compounds are already known to have antimicrobial activities. Ethyl acetate was already shown to have antibacterial properties [47,48], while 3-methylbutan-1-ol was shown to inhibit spore germination in *A. brasiliensis* [49] and development of the fungus *Guignarda citricarpa* [50], and 3-methylbutyl ethanoate was shown to inhibit the development of several yeasts and bacteria [49]. Several studies also reported antifungal properties of pentan-1-ol [51,52,53] and longifolene [53]. (+)-Sativene is known to be involved in plant growth promotion [54], but also for its phytotoxicity [55].

Interestingly, it was observed that low concentrations of 2-methylpropan-1-ol induced an increase of the growth of *C. sativus*, whereas high concentrations quickly inhibited growth (Figure 7A). The fact that some VOCs can induce opposite effects on the growth of a pathogen depending on the concentration applied has been shown for other compounds [56]. 2-methylpropan-1-ol is one of the most common VOCs emitted by microorganisms and could play a role in the ability of the colony to grow quickly in its environment (https://bioinformatics.charite.de/mvoc/, accessed on 2 May 2022). This compound has also been proven to be toxic to *C. albicans* cells [51].

In general, a difference in pigmentation accompanies the significant differences in growth observed for *F. culmorum*. This observation was already made in other studies studying the impact of VOCs from bacteria and yeast with antifungal properties [57]. A change in pigmentation could be a consequence of the modification of the functions of the fungal vacuole, an acidic organelle of fungi that controls cellular functions, and which would be damaged following the inhibitory effects [58,59].

We noted that the two fungal species can respond differently to the same individual VOCs at the same concentration (Figure 7), thus demonstrating different sensitivities and perhaps also variable mechanisms. For example, 3-methylbutan-1-ol reduced *C. sativus* growth with an increasing effect over time, while an increase in *F. culmorum* growth was observed on day 14 of contact. On the other hand, some VOCs, like ethyl acetate, 3-methylbutan-1-ol, and 2-methylpropan-1-ol, are emitted by both species and all cause mycelial growth reduction (at several levels) for both fungi. These three molecules have also been recorded to be emitted by other *Fusarium* species ([35,60,61]. This observation is interesting as it could be a first step in the discovery of common mechanisms of population control and the development of a broad-spectrum fungicide. In the same way, as both fungi belong to Ascomycota, it could be interesting to investigate other species of the same phylum to determine if common VOCs with interesting inhibition properties could be identified.

## 5. Conclusions

As stated before, QS in fungi, especially through VOCs, is still relatively unexplored. In our study, the volatolomes of *F. culmorum* and *C. sativus* were identified for the first time under low and high spore concentration conditions, with the second condition imitating overpopulation. New compounds were detected for both fungi when fungal species were placed in conditions of overpopulation. We have observed that the volatolomes emitted by the species in conditions of overpopulation had a negative impact on the mycelial growth, some of the emitted molecules possibly being QSMs. Among the emitted compounds, several were identified that slow growth, which could be linked to a possible QS effect. Analysis of genes inductions when the fungi are exposed to the promising VOCs could help to confirm this hypothesis.

Of course, a lot of research remains to be done, but we believe that the identification of these potential autoinducers is of prime importance to go deeper in the understanding of interactions between microorganisms, and that it paves the way to the development of new methods to control fungal populations, using sustainable alternatives to chemical pesticides.

## Figures and Tables

**Table 1:** List of individual VOCs and fungal species against which they were tested.

IUPAC Name	*F. culmorum*	*C. sativus*
(+)-Sativene		x
Longifolene		x
3-Methylbutyl ethanoate	x	
Pentan-1-ol	x	
Toluene	x	
2-Methylpropan-1-ol	x	x
3-Methylbutan-1-ol	x	x
Ethyl acetate	x	x

**Table 2:** VOCs emitted by *F. culmorum* and *C. sativus*.

									Emitted by			
									*F. culmorum*		*C. sativus*	
^a^	Compound name	CAS ^b^	Lit. ^c^	RIcal ^d^	RIstd ^e^	RIref ^f^	Identification ^g^	Ref RI ^h^	NC	O	NC	O
**Alcohol**	**2-Phenylethanol**	60-12-8	1, 2	1924		1924	MS, RI	NIST	7			
	**3-Methylbutan-1-ol**	123-51-3	2	1201	1201	1201	MS, RI, STD	NIST	7, 14	7, 14	7, 14	7, 14
	**2-Methylpropan-1-ol**	78-83-1	1	1062		1060	MS, RI	NIST	14	7, 14	14	7, 14
	**Pentan-1-ol**	71-41-0	1, 2	1270	1249	1260	MS, RI, STD	NIST		7		
	**Propan-1-ol**	71-23-8	1, 3	983		nd	MS, RI, STD	/				7, 14
**Alkene**	**2,6-Dimethylanisole**	22469-52-9	/	1397		nd	MS, RI	/	7			
	**Hemellitol**	526-73-8	/	1318		1320.6	MS, RI	NIST		7		
	**Isoprene**	78-79-5	5	nd		602	MS, RI	NIST			7, 14	7, 14
	**Mesitylene**	108-67-8	2	1247		1246	MS, RI	NIST	7, 14			
	**Pseudocumene**	95-63-6	2	1249		1249	MS, RI	NIST		14		
	**p-Xylene**	106-42-3	3	1066		1165	MS, RI	NIST	7, 14			
	**Toluene**	108-88-3	1	1021	1017	1020	MS, RI, STD	NIST	7, 14	7, 14		
**Alkane**	**Pentane**	109-66-0	3	nd		500	MS, RI	/			14	7, 14
**Esrer**	**3-Methylbutyl ethanoate**	123-92-2	1	1169	1186	1167	MS, RI, STD	NIST	7	7, 14		
	**Ethyl 2-methylbutyrate**	7452-79-1	1, 2	1022		1022	MS, RI	NIST				7, 14
	**Ethyl 3-methylbutanoate**	108-64-5	1, 2	1049		1049	MS, RI	NIST	14	14		
	**Ethyl acetate**	141-78-6	1, 3	888.5	901	900	MS, RI, STD	NIST	7, 14	7, 14		7, 14
**Ketone**	**Acetoin**	513-86-0	1	1285.9		1286	MS, RI	NIST				14
	**Butan-2-one**	78-93-3	1	nd		nd	MS, RI	/				7, 14
**Terpene**	**(-)-β-Acoradiene**	090457-37-7	2, 3, 5	1687.4		1693	MS, RI	NIST			7	7, 14
	**(+)-Aromadendrene**	489-39-4	1	1652.9		1650	MS, RI	Davies (1990)			7, 14	7
	**(+)-Sativene**	3650-28-0	1, 5	1524.5	1537	1527	MS, RI, STD	NIST			7, 14	7, 14
	**Cycloisosativene**	22469-52-9	/	1410		1483		Internal database			14	14
	**Epi-bicyclosesquiphellandrene**	54324-03-7	1, 3	1627.7		1633	MS, RI	NIST	7, 14	14		
	**Germacrene A**	75023-40-4	1, 5	1744.9		1745	MS, RI	NIST			7, 14	7, 14
	**Limonene**	3387-41-5	1, 3	1242		1234	MS, RI	pherobase	7	7		
	**Longifolene**	475-20-7	3, 5	1589.6	1600	1590	MS, RI, STD	NIST			7, 14	7, 14
	**Unkown sesquiterpene 1**	nd	/	nd		nd	MS, RI	/				7, 14
	**Unkown sesquiterpene 2**	nd	/	1674.4		nd	MS, RI	NIST			7	7
	**α-Chamigrene**	19912-83-5	1, 2	1661.7		nd	MS, RI	/	7, 14	14		
	**α-Gurjunene**	489-40-7	1	1535		1529		Internal database			7, 14	
	**β-Phellandrene**	555-10-2	3	1202.9		1202	MS, RI	NIST	7, 14			

^a^ Chemical family ^b^ CAS number (Chemical Abstracts Service) **^c^** Compound known in the literature to be emitted by 1 = fungi, 2 = *Fusarium* sp., 3 = *F. culmorum*, 4 = *Cochliobolus* sp., 5 = *C. sativus* (https://bioinformatics.charite.de/mvoc/index.php, accessed on 2 May 2022, [33,34,35]) ^d^ Kovats indices calculated on a VF-Wax polar capillary column with a homologous series of n-alkanes (C7-C30) ^e^ Kovats indices calculated for standards on a VF-Wax polar capillary column with a homologous series of n-alkanes (C7-C30) ^f^ Reference Kovats index in the literature ^g^ Identification proposal: MS, identification by comparison of spectral masses with Wiley275, pal600k and NBS75K databases; RI: identification by retention indices with literature data; STD: comparison with retention times and spectral masses of available standards ^h^ Reference RI, NC: normal growth conditions, O: Overpopulation conditions of growth. 7 and 14 indicate the number of days of growth when the VOC was detected.

## Data Availability

Not applicable.
